# Reversal of Alpha-Synuclein Fibrillization by Protein Disulfide Isomerase

**DOI:** 10.3389/fcell.2020.00726

**Published:** 2020-07-30

**Authors:** Albert Serrano, Xin Qiao, Jason O. Matos, Lauren Farley, Lucia Cilenti, Bo Chen, Suren A. Tatulian, Ken Teter

**Affiliations:** ^1^Burnett School of Biomedical Sciences, College of Medicine, University of Central Florida, Orlando, FL, United States; ^2^Department of Physics, College of Sciences, University of Central Florida, Orlando, FL, United States

**Keywords:** amyloid, chaperone, disaggregase, ERp57, neurodegeneration

## Abstract

Aggregates of α-synuclein contribute to the etiology of Parkinson’s Disease. Protein disulfide isomerase (PDI), a chaperone and oxidoreductase, blocks the aggregation of α-synuclein. An S-nitrosylated form of PDI that cannot function as a chaperone is associated with elevated levels of aggregated α-synuclein and is found in brains afflicted with Parkinson’s Disease. The protective role of PDI in Parkinson’s Disease and other neurodegenerative disorders is linked to its chaperone function, yet the mechanism of neuroprotection remains unclear. Using Thioflavin-T fluorescence and transmission electron microscopy, we show here for the first time that PDI can break down nascent fibrils of α-synuclein. Mature fibrils were not affected by PDI. Another PDI family member, ERp57, could prevent but not reverse α-synuclein aggregation. The disaggregase activity of PDI was effective at a 1:50 molar ratio of PDI:α-synuclein and was blocked by S-nitrosylation. PDI could not reverse the aggregation of malate dehydrogenase, which indicated its disaggregase activity does not operate on all substrates. These findings establish a previously unrecognized disaggregase property of PDI that could underlie its neuroprotective function.

## Introduction

Protein disulfide isomerase (PDI) is mainly located in the endoplasmic reticulum (ER) but also operates at other intracellular and extracellular locations (Turano et al., 2002; Ali Khan and Mutus, 2014; Soares and Laurindo, 2017). It is a U-shaped protein with an abb′xa′ organization that contains four thioredoxin domains and a flexible x linker. The inactive b and b′ thioredoxin domains at the base of the U are primarily involved with substrate binding, while the a and a′ domains at each arm of the U act as oxidoreductases (Wang et al., 2015; Freedman et al., 2017). Several other PDI family members such as ERp57 also reside in the ER and, like PDI, have a modular structure of repeated thioredoxin domains (Ellgaard and Ruddock, 2005). In addition to their oxidoreductase activity, PDI and its related family members can function as chaperones to prevent the aggregation of misfolded proteins. This includes proteins that lack cysteine residues such as α-synuclein (Cai et al., 1994; Song and Wang, 1995; Cheng et al., 2010; Ranjan and Kumar, 2016), an amyloid-forming protein implicated in Parkinson’s Disease (Bisaglia et al., 2009).

A dysfunctional, S-nitrosylated form of PDI (SNO-PDI) that cannot prevent protein aggregation is commonly associated with amyloid formation and neurodegeneration (Andreu et al., 2012; Halloran et al., 2013; Conway and Harris, 2015). The loss of PDI function resulting from its S-nitrosylation leads to the formation of α-synuclein fibrils in neuronal cell culture and organotypic brain slices (Kabiraj et al., 2014; Wu et al., 2014; Xu et al., 2014). Furthermore, SNO-PDI has been identified in brains from patients with Parkinson’s Disease but not from healthy brains (Uehara et al., 2006). Despite its protective role against Parkinson’s Disease and other neurodegenerative disorders, the mechanism by which PDI blocks amyloid formation remains unclear.

The interaction between PDI and amyloid fibrils may mimic the interaction between PDI and the multimeric cholera toxin (CT). PDI displaces the A1 subunit of CT from its non-covalent association with the rest of the toxin, which is a prerequisite for cellular toxin activity (Tsai et al., 2001; Taylor et al., 2011; Heggelund et al., 2015). ERp57 and ERp72, two other ER-localized PDI family members, cannot disassemble CT (Taylor et al., 2014). This function is unique to PDI, which explains why PDI-deficient cells are resistant to CT (Taylor et al., 2011). We hypothesized that PDI could disrupt protein aggregation by dislodging individual proteins from an aggregate in the same way it breaks apart CT. Such a process could also allow PDI to reverse, as well as prevent, protein aggregation. This would place PDI in the family of protein “disaggregases” (Glover and Lindquist, 1998; Doyle et al., 2013; Torrente and Shorter, 2013; Nillegoda and Bukau, 2015). Here, we report that PDI completely reverses α-synuclein fibrillization at a 1:10 molar ratio of PDI:substrate and partially reverses fibrillization at a 1:50 molar ratio. This is the first time PDI has been shown to dissolve amyloid fibrils. Nascent but not mature fibrils of α-synuclein were affected by the disaggregase activity of PDI. The dissolution of α-synuclein fibrils did not occur when PDI function was disrupted by S-nitrosylation or treatment with PDI inhibitors. Our observations thus establish a novel and substrate-specific disaggregase function for PDI that provides a possible basis for its neuroprotective role in Parkinson’s Disease.

## Materials and Methods

### Protein Preparations

Human PDI with an N-terminal hexahistidine tag was purified from *Escherichia coli* strain BL21(DE3)pLysS using Talon metal affinity chromatography as previously described (Zheng et al., 2016). PDI purified from bovine liver was purchased from Sigma-Aldrich (St. Louis, MO, United States), human ERp57 was purchased from Novus Biologicals (Littleton, CO, United States), and bovine serum albumin (BSA) was purchased from Fisher Scientific (Hampton, NH, United States). The E46K mutant of α-synuclein (rPeptide, Bogart, GA, United States) was dissolved in hexafluoroisopropanol (Sigma-Aldrich) at a final concentration of 1 mg/mL. After incubation for 60 min at room temperature, the solution was placed at −20°C overnight. The thawed solution was dried under a stream of nitrogen gas, subjected to vacuum desiccation for 60 min, and resuspended in 1 mL of 20 mM Tris–HCl (pH 7.4) containing 100 mM NaCl. To initiate aggregation, 70 μM of α-synuclein was vortexed for 1 s and transferred in a 250 μL volume to a 4 × 4 mm quartz cuvette (Starna Cells, Atascadero, CA, United States) containing a stir bar. Samples were incubated at 37°C with constant shaking at 600 rpm.

### Thioflavin-T (ThT) Aggregation Assay

Samples of α-synuclein were mixed with a final concentration of 20 μM ThT (Anaspec, Inc., Fremont, CA, United States) in a buffer of 20 mM Tris–HCl (pH 7.4) containing 100 mM NaCl. When indicated, PDI, ERp57, or BSA were also added to α-synuclein. At defined intervals after the initiation of aggregation, ThT fluorescence spectra were recorded from 430 to 540 nm with excitation at 450 nm, using a Jasco (Easton, MD, United States) J-810 spectropolarimeter with a fluorescence attachment. Maximum ThT fluorescence at 482 nm was used for data analysis.

### Transmission Electron Microscopy (TEM)

Samples of α-synuclein taken 0, 18, 30, or 54 h after the initiation of aggregation were transferred in a 5 μL volume to a TEM grid (Ted Pella Inc., Redding, CA, United States). After 5 min, 2 μL of 3% uranyl acetate was added to the grid. This was followed by four washes with 5 μL ddH_2_0, placement of the grid in a sample holder, and insertion of the sample holder into the JEOL (Peabody, MA, United States) 1011 transmission electron microscope operated at 80 keV. Images were captured at 8,000× magnification.

### Drug Treatments

Protein disulfide isomerase was exposed for 30 min at room temperature to 1 mM S-nitrosylated cysteine, 50 μM ribostamycin (Sigma-Aldrich), or 1 mM quercetin-3-rutinoside (Q3R) (Sigma-Aldrich). SNO-cysteine was freshly prepared by mixing 100 mM L-cysteine and 100 mM NaNO_2_, followed by acidification with 5% (v/v) 10 N HCl. A PDI stock concentration of 1 mg/mL was used for all treatments.

### Malate Dehydrogenase (MDH) Aggregation Assay

A quartz cuvette containing 250 μL of 3 μM MDH in 50 mM sodium phosphate buffer (pH 7.4) was placed in a Jasco J-810 spectropolarimeter with a Peltier temperature controller and an additional photomultiplier tube attached at a right angle that can be used for fluorescence or light scattering measurements. Aggregation was induced by heating the sample within the instrument to 45°C. Various concentrations of PDI were also added to the sample, either before the onset of aggregation (0 min) or after 10 min of aggregation. Right angle light scattering at 510 nm was recorded every minute for 24 min, which typically represented the plateau of MDH aggregation. Experimental values were corrected for the signal from buffer alone or, for MDH + PDI samples, buffer containing only PDI (which produced a minimal signal). Background-subtracted values were then expressed as percentages of the maximum light scattering value for the experiment.

## Results

### The Disaggregase Property of PDI

**FIGURE 1:**
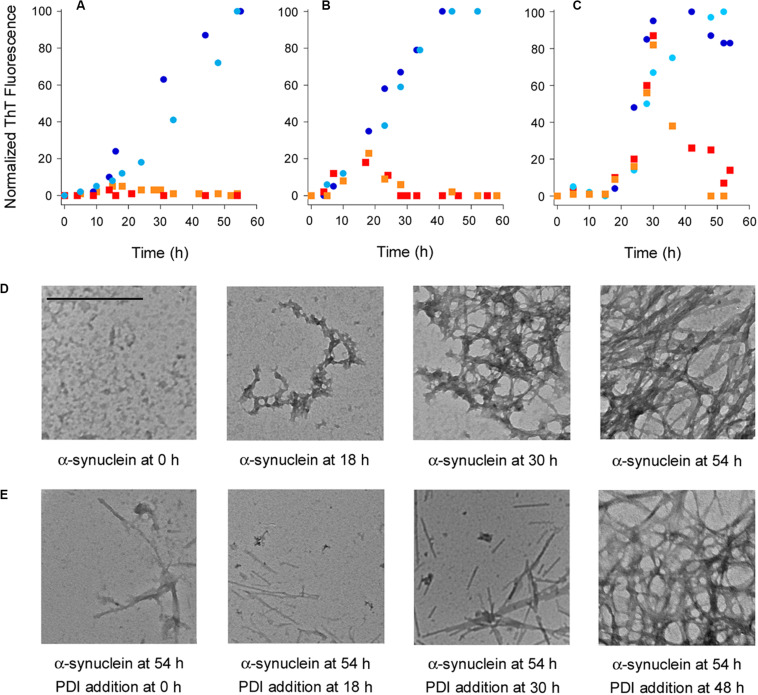
Protein disulfide isomerase (PDI) prevents and reverses the aggregation of α-synuclein. To initiate fibril formation at 0 h, α-synuclein mutant E46K was diluted to 70 μM at 37°C with constant agitation in a buffer of 20 mM Tris–HCl (pH 7.4) containing 100 mM NaCl. **(A–C)** Fibril formation was monitored by ThT fluorescence at 482 nm, which was normalized to the maximal ThT signal recorded for untreated α-synuclein over the course of the experiment. Circles represent untreated α-synuclein and squares represent α-synuclein samples incubated with 7 μM PDI at **(A)** 0 h, **(B)** 18 h, or **(C)** 30 h after the initiation of fibrillization. Dark blue circles and red squares represent matched samples from one experiment; light blue circles and orange squares represent matched samples from a second experiment. **(D)** TEM images of α-synuclein at 8,000× magnification were taken at the indicated times after the initiation of fibrillization. **(E)** TEM images of α-synuclein at 8,000× magnification were taken 54 h after the initiation of fibrillization. PDI (7 μM) was added to α-synuclein at the indicated times after the initiation of fibrillization. Scale bar represents 500 nm for all images.

Protein disulfide isomerase is known to inhibit the aggregation of α-synuclein (Cheng et al., 2010; Ranjan and Kumar, 2016), but the potential dissolution of α-synuclein fibrils by PDI has not been tested. ThT and TEM were used to examine this possibility. Our studies used the E46K mutant of α-synuclein that is linked to familial cases of Parkinson’s Disease and aggregates faster than the wild-type protein (Zarranz et al., 2004; Greenbaum et al., 2005). ThT is a fluorophore that undergoes fluorescence enhancement and red shift upon contact with amyloid fibrils (Biancalana and Koide, 2010). As shown in Figures 1A–C (circles), we recorded a time-dependent increase in the ThT signal resulting from α-synuclein aggregation. No ThT signal was detected over the course of the experiment when PDI was added to α-synuclein at the beginning of the experiment (Figure 1A, squares). This demonstrated PDI can prevent the aggregation of α-synuclein and was consistent with previous reports (Cheng et al., 2010; Ranjan and Kumar, 2016). However, the reversal of α-synuclein fibril formation by PDI has not been previously reported. We found, for the first time, that PDI not only inhibits the initiation of α-synuclein fibrillization but efficiently reverses the ongoing aggregation process when added to α-synuclein fibrils 18 h (Figure 1B, squares) or 30 h (Figure 1C, squares) after the initiation of aggregation. In both cases, the ThT signal resulting from α-synuclein aggregation increased in parallel with the untreated control sample until PDI was added. The ThT signal then dropped to the background level of fluorescence within 12 (Figure 1B) to 24 h (Figure 1C) of exposure to PDI. These data uncover a novel property of PDI: its ability to dissolve nascent α-synuclein fibrils at a sub-stoichiometric 1:10 molar ratio of PDI:substrate.

Transmission electron microscopy visualized the time-dependent aggregation of α-synuclein, with increasing fibrillization seen over the course of 54 h (Figure 1D). However, few fibrils were seen after 54 h when PDI was added to α-synuclein following 0, 18, or 30 h of aggregation (Figure 1E). The amyloid structures initially present at 18 or 30 h of aggregation were thus broken down by the subsequent addition of PDI and were largely absent from the sample by the end of the 54 h experiment. Most fields of view were blank under these conditions, but residual fibrils were occasionally detected and are shown in order to acknowledge the presence of some aggregated α-synuclein. This minimal level of fibrillization did not generate a significant ThT signal (Figures 1A–C) and was consistent with a previous report that used atomic force microscopy to record the near-complete inhibition of α-synuclein aggregation by PDI (Ranjan and Kumar, 2016). The mostly linear aggregates of α-synuclein that were present after PDI addition appeared to be structurally distinct from the fibrils generated by untreated α-synuclein. However, it is possible that the lack of cross-branched fibrils in PDI-treated samples simply reflects the lower density of fibrils under these conditions. Additional work will be required to understand the potential structural differences between untreated and PDI-treated fibrils of α-synuclein.

Fibrillization was not affected when PDI was added to a 48 h aggregate of α-synuclein. In this case, the dense accumulation of α-synuclein fibrils was still present at the end of the experiment (Figure 1E, far right panel). Likewise, the ThT signal recorded for α-synuclein fibrils after 48 h of aggregation did not decrease when PDI was added for an additional 6 h: the ThT signal at 54 h from α-synuclein with PDI was 108 ± 11% (*n* = 3, standard deviation) of the ThT signal at 48 h from α-synuclein alone. Extending the interval after PDI exposure by an additional 18 h did not decrease the ThT signal recorded at 48 h for α-synuclein alone. These observations indicated that PDI can reverse α-synuclein aggregation at its early and intermediate stages but cannot disrupt the final, dense meshwork of α-synuclein fibrils that are thought to be neuroprotective (Uversky, 2007).

### PDI Functions as a Disaggregase at Sub-Stoichiometric Molar Ratios of PDI:Substrate

**FIGURE 2:**
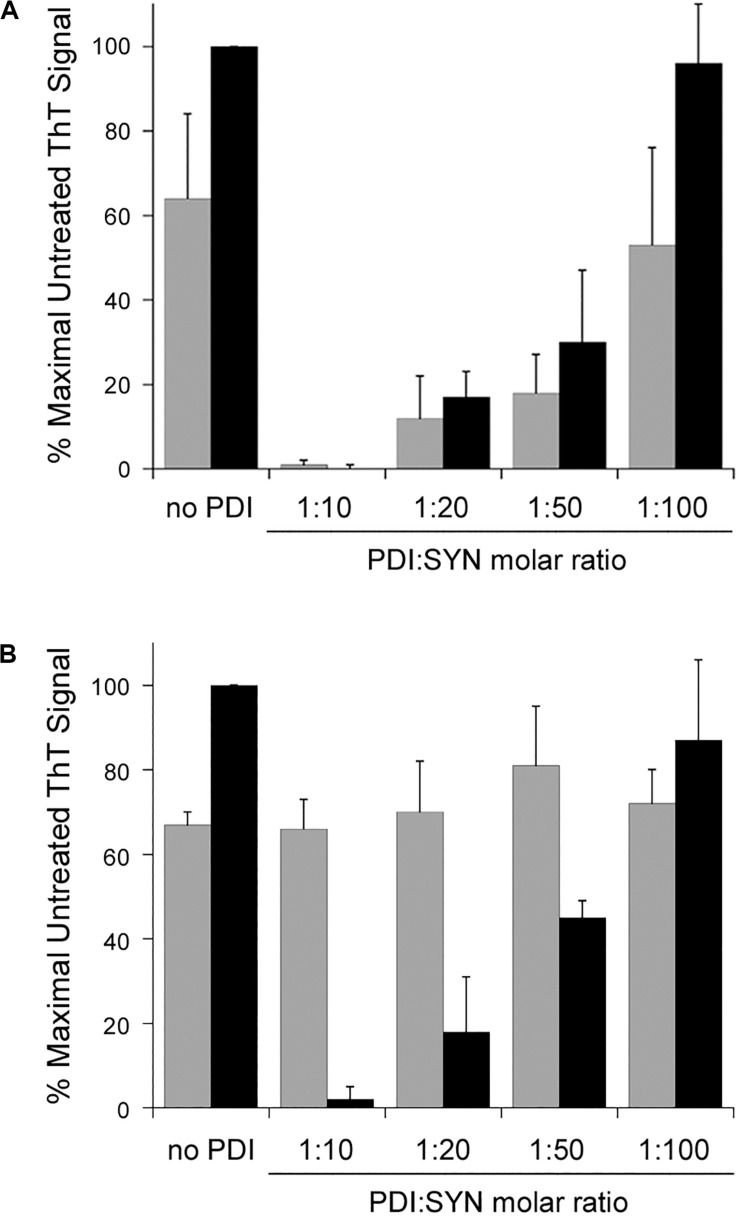
Protein disulfide isomerase (PDI) disaggregase activity is effective at sub-stoichiometric molar ratios. To initiate fibril formation at 0 h, α-synuclein mutant E46K was diluted to 70 μM at 37°C with constant agitation. Data represent the averages ± standard deviations of three independent experiments. **(A)** PDI was mixed at the indicated molar ratios with α-synuclein at 0 h. ThT fluorescence at 482 nm was recorded 30 h (gray bars) or 52 h (black bars) after the initiation of fibrillization. **(B)** Samples of α-synuclein were allowed to aggregate for 30 h. After recording ThT fluorescence at 30 h (gray bars), the indicated molar ratios of PDI were added to the sample for another 22 h before a second ThT measurement was taken (black bars).

Our initial experiments were performed with a 1:10 molar ratio of PDI:α-synuclein. Subsequent titration experiments documented a substantial inhibition of α-synuclein aggregation at 1:20 and 1:50 molar ratios of PDI:α-synuclein. Only a minor inhibitory effect was obtained with a 1:100 ratio of PDI:α-synuclein. These results were observed after 30 h of aggregation (Figure 2A, gray bars). When the assay was extended to 52 h (Figure 2A, black bars), we found PDI was less effective at blocking α-synuclein aggregation when present at 1:50 or 1:100 molar ratios. In fact, no inhibitory effect was recorded for the 1:100 ratio of PDI:α-synuclein. A previous ThT study that used a single 48 h end-point measurement to monitor the impact of PDI on α-synuclein aggregation did not report a substantial inhibitory effect with a 1:100 molar ratio of PDI:α-synuclein and documented an ∼40% inhibition of α-synuclein aggregation with a 1:50 molar ratio of PDI:α-synuclein (Cheng et al., 2010). Our measurements were consistent with these observations and further demonstrated that low quantities of PDI can have transient inhibitory effects on α-synuclein aggregation.

Titration experiments also identified the minimal quantity of PDI required for its disaggregase activity. All samples of α-synuclein were allowed to aggregate for 30 h in the absence of PDI (Figure 2B, gray bars). Various amounts of PDI were then added for an additional 22 h, which was followed by a second ThT measurement on the same sample of α-synuclein (Figure 2B, black bars). We found that PDI could not reverse α-synuclein aggregation at a 1:100 molar ratio but could partially dissolve aggregated α-synuclein at a 1:50 molar ratio. This effect was dose-dependent, with a greater level of disaggregation at a 1:20 molar ratio and complete disaggregation at a 1:10 ratio. Thus, both the prevention and reversal of α-synuclein fibrillization can occur with low, sub-stoichiometric quantities of PDI.

### SNO-PDI and ERp57 Do Not Function as Disaggregases

**FIGURE 3:**
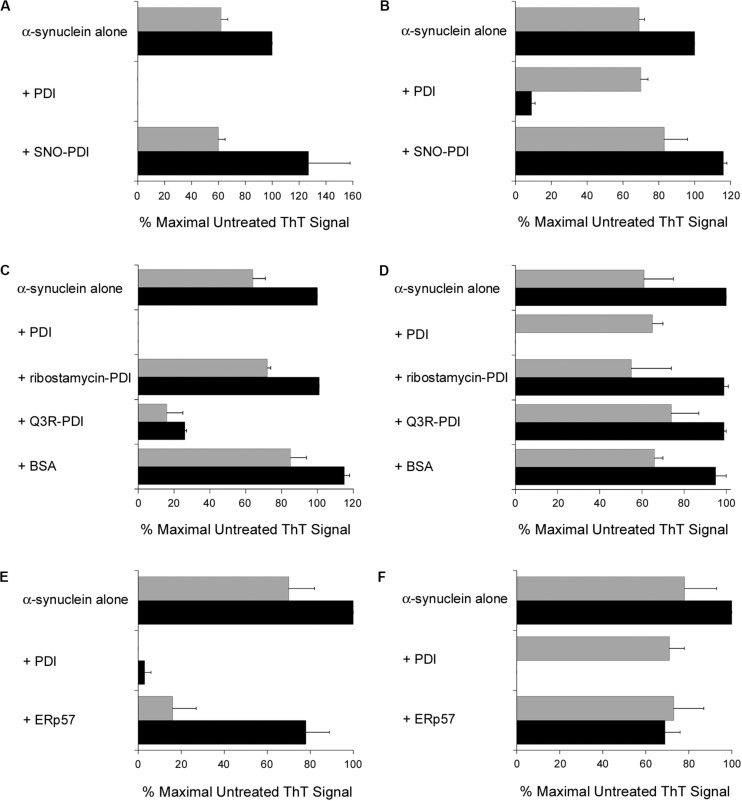
Specificity of the protein disulfide isomerase (PDI) disaggregase activity. To initiate fibril formation at 0 h, α-synuclein mutant E46K was diluted to 70 μM at 37°C with constant agitation. Data represent the averages ± ranges of two independent experiments. **(A)** PDI or SNO-PDI was mixed at 7 μM final concentration with α-synuclein at 0 h. ThT fluorescence was recorded 30 h (gray bars) or 52 h (black bars) after the initiation of fibrillization. **(B)** Samples of α-synuclein were allowed to aggregate for 30 h. After recording ThT fluorescence at 30 h (gray bars), 7 μM of PDI or SNO-PDI was added to the sample for another 22 h before a second ThT measurement was taken (black bars). **(C)** 7 μM PDI, 7 μM ribostamycin-treated PDI, 7 μM Q3R-treated PDI, or 10 μM BSA was mixed with α-synuclein at 0 h. ThT fluorescence was recorded 30 h (gray bars) or 52 h (black bars) after the initiation of fibrillization. **(D)** Samples of α-synuclein were allowed to aggregate for 30 h. After recording ThT fluorescence at 30 h (gray bars), 7 μM PDI, 7 μM ribostamycin-treated PDI, 7 μM Q3R-treated PDI, or 10 μM BSA was added to the sample for another 22 h before a second ThT measurement was taken (black bars). **(E)** PDI or ERp57 was mixed at a 1:10 molar ratio with α-synuclein at 0 h. ThT fluorescence at 482 nm was recorded 30 h (gray bars) or 52 h (black bars) after the initiation of fibrillization. **(F)** Samples of α-synuclein were allowed to aggregate for 30 h. After recording ThT fluorescence at 30 h (gray bars), a 1:10 molar ratio of PDI or ERp57 was added to the sample for another 22 h before a second ThT measurement was taken (black bars).

Additional ThT experiments demonstrated that a functional PDI is required for the disruption of α-synuclein fibrillization. The chaperone as well as oxidoreductase function of PDI is blocked by S-nitrosylation (Uehara et al., 2006), and we found SNO-PDI could neither inhibit (Figure 3A) nor reverse (Figure 3B) the aggregation of α-synuclein. Two other treatments also blocked the disruptive effect of PDI on α-synuclein aggregation. Ribostamycin is an aminoglycoside antibiotic that specifically inactivates the chaperone function of PDI (Horibe et al., 2001; Ko and Kay, 2004). It prevents the PDI-driven disassembly of CT (Taylor et al., 2014), and, as shown here, also prevented PDI from both inhibiting (Figure 3C) and reversing (Figure 3D) the aggregation of α-synuclein. In contrast, Q3R-treated PDI could partially inhibit α-synuclein aggregation (Figure 3C) but did not dissolve a 30 h aggregate of α-synuclein (Figure 3D). Q3R is an established PDI inhibitor (Jasuja et al., 2012) that prevents the PDI-driven disassembly of CT (Guyette et al., 2019). The ability of Q3R-treated PDI to block but not reverse α-synuclein aggregation suggests the disaggregase property of PDI involves a mechanism that is distinct from its ability to prevent aggregation. BSA did not block (Figure 3C) or reverse (Figure 3D) the aggregation of α-synuclein, which further documented the specific disruptive effect of PDI on α-synuclein aggregation.

It should be noted that the experiments of Figures 3C,D were performed with PDI purified from bovine liver, while all other experiments in this work were performed with recombinant human PDI. Both preparations of PDI yielded the same result, so the disaggregase activity of PDI could not be attributed to an artifact or contaminant from our purification procedure for recombinant PDI.

ERp57, another ER-localized oxidoreductase with a similar structural organization to PDI, is also linked to neurodegenerative diseases (Hettinghouse et al., 2018). We found that ERp57 could also block the fibrillization of α-synuclein after at least 30 h from the onset of aggregation, albeit with less efficacy than PDI (Figure 3E). However, in contrast to PDI, ERp57 could not dissolve nascent aggregates of α-synuclein at a 1:10 molar ratio of ERp57:α-synuclein (Figure 3F). ERp57 prevented further amyloid formation when added to α-synuclein after 30 h of aggregation, but it did not break down the existing fibrils. Thus, the disaggregase property of PDI is not shared by the entire PDI family of proteins.

### PDI Does Not Dissolve All Protein Aggregates

**FIGURE 4:**
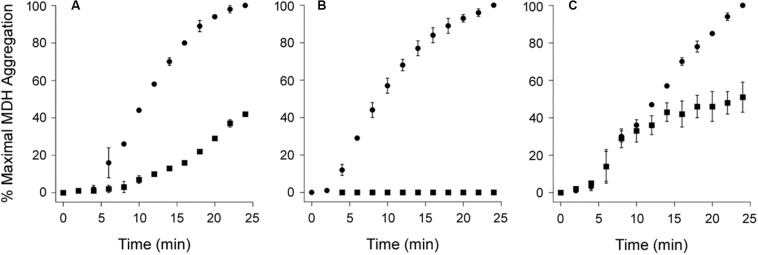
Protein disulfide isomerase (PDI) does not reverse the aggregation of malate dehydrogenase (MDH). At time point 0, the aggregation of MDH was initiated by heating the sample to 45°C with stirring. Aggregation was monitored by light scattering. Data represent the averages ± ranges of two independent experiments. **(A,B)** MDH was left untreated (circles) or was co-incubated with PDI (squares) for the duration of the assay at **(A)** 1:1 or **(B)** 3:1 molar ratios of PDI:MDH. **(C)** After 10 min of aggregation, MDH was left untreated (circles) or was supplemented with PDI (squares) for the remainder of the assay at a 7:1 molar ratio of PDI:MDH.

As shown in Figure 4, PDI does not exhibit a disaggregase activity against all substrates. We found its effect on the aggregation of MDH resembled a standard chaperone-substrate interaction in which a molar excess of PDI over substrate was required to completely inhibit the aggregation of MDH (Figures 4A,B). Moreover, PDI could not reverse the aggregation of MDH when added in 7-fold molar excess over MDH (Figure 4C). MDH is used as a model aggregation-prone substrate for the activity of molecular chaperones such as PDI (Cheung and Churchich, 1999; Goloubinoff et al., 1999; Nillegoda et al., 2015), but it does not form amyloid structures. The aggregation of MDH is therefore detected by light scattering instead of ThT fluorescence. Collectively, our data indicate the disaggregase activity of PDI is restricted to select substrates such as CT and α-synuclein.

## Discussion

Fibrils of α-synuclein form cytoplasmic Lewy Bodies, a hallmark of Parkinson’s Disease (Gallegos et al., 2015). However, oligomers of α-synuclein can also be found in the ER (Bellucci et al., 2011; Colla et al., 2012a, b, 2017) and the extracellular space where they promote a prion-like transmission to healthy neurons (Steiner et al., 2011; Brundin et al., 2015; Gallegos et al., 2015). PDI may interact with α-synuclein at any of these sites: although it is primarily found in the ER, PDI also functions at other intracellular and extracellular locations (Turano et al., 2002; Ali Khan and Mutus, 2014; Soares and Laurindo, 2017). Our data suggest the neuroprotective function of PDI is related to its disaggregase property that may be active in both intracellular and extracellular environments. SNO-PDI does not break down nascent amyloid fibrils (Figures 3A,B), which helps explain its inability to disrupt α-synuclein aggregation in brain tissue and neuronal cell culture (Kabiraj et al., 2014; Wu et al., 2014; Xu et al., 2014) as well as why SNO-PDI is found in brains afflicted with Parkinson’s Disease (Uehara et al., 2006). The inactivation of PDI through S-nitrosylation is a common feature of neurodegenerative disorders (Andreu et al., 2012; Halloran et al., 2013; Conway and Harris, 2015), so our study provides a possible explanation for the general effect of PDI dysfunction on neurodegeneration.

We identified three conditions that blocked the *in vitro* dissolution of α-synuclein fibrils by PDI: S-nitrosylation, Q3R treatment, and ribostamycin treatment. The first two conditions disrupt both the oxidoreductase and chaperone activities of PDI, but ribostamycin only inhibits the chaperone activity of PDI. The effects of PDI on α-synuclein aggregation thus appear to be related to its chaperone activity, which is consistent with the general function of a chaperone in preventing protein aggregation. However, based upon our results with Q3R, the two chaperone-linked functions of PDI (inhibition and disaggregation of α-synuclein fibrils) appear to involve distinct mechanisms. Q3R-treated PDI could thus prevent but not reverse the aggregation of α-synuclein.

There are several similarities between the dissolution of α-synuclein fibrils by PDI and the PDI-driven disassembly of CT. Both events are inhibited when PDI is treated with Q3R or ribostamycin (Figure 3D; Taylor et al., 2014; Guyette et al., 2019). Both events also appear to specifically require PDI, as ERp57 cannot disassemble CT (Taylor et al., 2014) and does not reverse α-synuclein fibril formation (Figure 3F). We have proposed that the PDI-driven disassembly of CT involves a physical mechanism in which the partial unfolding of PDI that occurs upon contact with CTA1 acts as a wedge to push CTA1 away from the rest of the toxin (Taylor et al., 2014). In support of this model, PDI did not disassemble CT when its substrate-induced unfolding was blocked by Q3R or ribostamycin (Taylor et al., 2014; Guyette et al., 2019). Furthermore, ERp57 did not unfold when bound to CTA1 and did not release CTA1 from the rest of the toxin (Taylor et al., 2014). These collective observations suggest the disaggregase property of PDI is linked to a unique structural alteration that occurs upon contact with select substrates such as CTA1 or α-synuclein.

S-nitrosylated form of PDI may also contribute to neurodegeneration through a toxic unfolded protein response (Perri et al., 2016). In this case, the accumulation of misfolded secretory proteins resulting from the loss of PDI function would lead to ER stress, chronic activation of the unfolded protein response, and apoptosis. This effect and the loss of PDI disaggregase activity against α-synuclein are not mutually exclusive; both could play a role in neurodegeneration. However, the disaggregase model presents a clear therapeutic strategy: PDI functions in the extracellular environment (Turano et al., 2002; Ali Khan and Mutus, 2014; Soares and Laurindo, 2017) and could therefore be applied as a novel, non-immunogenic therapeutic agent to clear nascent fibrils of extracellular α-synuclein that may propagate the disease through prion-like transmission (Brundin et al., 2015). The extracellular, ATP-independent function of PDI also meets two key criteria for the development of a therapeutic disaggregase to treat neurodegenerative diseases (Shorter, 2017). Our work sets the foundation for such studies, as well as future work to define the structural basis of PDI-driven disaggregation.

## Data Availability Statement

All datasets presented in this study are included in the article/supplementary material.

## Author Contributions

AS, XQ, JM, LF, and LC performed the experiments. KT conceptualized the study and wrote the manuscript. AS, BC, ST, and KT analyzed the data. BC and ST edited the manuscript. All authors contributed to the article and approved the submitted version.

## Conflict of Interest

The authors declare that the research was conducted in the absence of any commercial or financial relationships that could be construed as a potential conflict of interest.
